# Effect of adaptive abilities on utilities, direct or mediated by mental health?

**DOI:** 10.1186/1477-7525-8-130

**Published:** 2010-11-12

**Authors:** Yvette Peeters, Adelita V Ranchor, Thea PM Vliet Vlieland, Anne M Stiggelbout

**Affiliations:** 1 Department of Medical Decision Making, Leiden University Medical Centre, Leiden, The Netherlands; 2 Graduate School of Medical Sciences, Department of Health Science, University Medical Centre Groningen, University of Groningen, Groningen, The Netherlands; 3 Department of Rheumatology, Leiden University Medical Centre, Leiden, The Netherlands

## Abstract

**Background:**

In cost-utility analyses gain in health can be measured using health state utilities. Health state utilities can be elicited from members of the public or from patients. Utilities given by patients tend to be higher than utilities given by members of the public. This difference is often suggested to be explained by adaptation, but this has not yet been investigated in patients. Here, we investigate if, besides health related quality of life (HRQL), persons' ability to adapt can explain health state utilities. Both the direct effect of persons' adaptive abilities on health state utilities and the indirect effect, where HRQL mediates the effect of ability to adapt, are examined.

**Methods:**

In total 125 patients with Rheumatoid Arthritis were interviewed. Participants gave valuations of their own health on a visual analogue scale (VAS) and time trade-off (TTO). To estimate persons' ability to adapt, patients filled in questionnaires measuring Self-esteem, Mastery, and Optimism. Finally they completed the SF-36 measuring HRQL. Regression analyses were used to investigate the direct and mediated effect of ability to adapt on health state utilities.

**Results:**

Persons' ability to adapt did not add considerably to the explanation of health state utilities above HRQL. In the TTO no additional variance was explained by adaptive abilities (Δ R^2 ^= .00, β = .02), in the VAS a minor proportion of the variance was explained by adaptive abilities (Δ R^2 ^= .05, β = .33). The effect of adaptation on health state utilities seems to be mediated by the mental health domain of quality of life.

**Conclusions:**

Patients with stronger adaptive abilities, based on their optimism, mastery and self-esteem, may more easily enhance their mental health after being diagnosed with a chronic illness, which leads to higher health state utilities.

## Background

In health care, decisions are made about treatment at the level of individual patients, of patient groups (guideline development), and at the societal level [1]. Decisions about guideline development and decisions at the societal level are often guided by cost-utility analyses. In these analyses the gain in health obtained by treatment is compared with the costs that have to be made in order to obtain this gain [2]. To assess the value of this gain, cost-utility analyses make use of health state valuations, i.e. health state utilities.

A health state utility is a preference for a particular health state compared with perfect health and immediate death. Utilities can be seen as a global valuation of health related quality of life (HRQL) [3] and can be expected to show a strong relationship with health status. Nevertheless, only between 18% and 43% of the variance in health state utilities can be explained by HRQL. Most of the variance still remains unexplained [4].

Health state utilities can be elicited from members of the public and from patients. Members of the public tend to give lower health state valuations, compared to patients [5]. This discrepancy in health state valuations has, among others, been suggested to be explained by the failure of members of the public to anticipate on their ability to adapt. Patients adapt to the physical and psychological challenges of their illness [6]. When health state valuations are elicited from patients, some of the variance in health state utilities might be explained by this adaptation [7-9].

Tentative support has been found for the effect of adaptation on health state valuations. Members of the public who were made aware of their ability to adapt gave higher valuations on a person trade-off (PTO) and on a visual analogue scale (VAS) measuring quality of life [10,11], but not on the time trade-off (TTO) nor on the standard gamble (SG) [12]. Whether health state utilities given by patients are actually correlated with adaptation has not been topic of study yet.

Adaptation can be defined as a response that diminishes or remains the same despite constant or increasing stimulus levels [13]. The outcome of adaptation can be measured by change over time, such as change in well-being [14] or life satisfaction [15,16]. If researchers aim to gain more insight in the process of adaptation itself, adaptation can be conceptualised through certain coping-strategies [17,18]. These coping-strategies are, among others, enabled by personal resources.

By studying adaptation Taylor [19] developed the Cognitive Adaptation Theory (CAT) which is based on cognitive interviews with chronically ill persons. This theory is one of the dominant theories in health psychology and has often been used to empirically test adaptation. Research using this theory suggests that psychological adjustment to an illness occurs around four themes; a search for meaning in the experience, an attempt to regain mastery over the event and over one's life more generally, an effort to enhance one's self-esteem, and the ability to find positive illusions, i.e. optimism. These concepts as suggested in the CAT are further described below.

After a threatening event, people often cannot find a sense of meaning in the experience and lose their feelings of mastery and of self-esteem. Most people manage to re-establish these over time. According to Taylor, this re-establishment is based on so-called positive illusions. People develop unrealistic beliefs that make it possible to regain control over the event and over one's life and to regain self-esteem [19]. Although positive illusions may create unrealistic and maybe 'false' ideas, these illusions have been found to be important resources [20].

Previous studies have shown that patients who score high on indicators of CAT have better psychological functioning [21-24], they are less anxious and depressed, report more vitality and have a better mental functioning [22,25,26]. Moreover, patients with a higher score on indicators of CAT reported better physical functioning [22,23], they showed fewer new coronary events or hospital admissions [21,26] and lived longer [27]. It thus appears that patients who have higher self-esteem, mastery, and optimism, and who find a meaning in the experience have better abilities to adapt.

No standard method is available for investigating the ability to adapt based on CAT. Studies have used different indicators and methods for their analyses. For instance, studies have included indicators measuring optimism, mastery and self-esteem, but often exclude finding meaning. To our knowledge, only in two studies the effect of finding meaning was included [27,28]. The rationale to exclude benefit finding was described by Major et al. [29] and Chan et al. [23]. Both research groups suggest that mastery, self-esteem, and optimism are stable personality traits representing a persons' resilience, whereas finding meaning might be seen as a process facilitated by these personality traits.

Apart from this variety of indicators of CAT included to measure adaptive abilities, studies have also used different ways to measure these indicators. Some studies have analysed the effects of the different indicators separately [25,30], some have created a scale taking the indicators together [26-28,31], and again others have investigated each indicator separately as well as an aggregate scale of the indicators together [21-23]. The latter studies revealed that besides the effect of the aggregate scale, often only one of the indicators had an effect on the outcome measurement. Since the overall results of these studies show different 'single' indicators to reveal an effect, indicators of persons' abilities to adapt cannot be simplified to one single indicator. Exploring the results of these studies further, it seems that significant effects have mostly been seen in studies using an aggregate scale. Therefore, in the present study persons' ability to adapt is constructed with an aggregate scale based on mastery, self-esteem and optimism.

The first aim of this study was to investigate if above HRQL, persons' adaptive abilities explain health state utilities. That is:

Do adaptive abilities account for the unexplained variance in health state utilities above the variance explained by HRQL?

Another possibility is that adaptation, in this study measured through persons' ability to adapt, has an indirect effect on utilities, through HRQL. As described above, adaptive abilities does affect psychological and physical functioning [26]. This would fit the hierarchical model of Spilker and Revicki [32], in which three levels of quality of life are distinguished that have mutual impact on each other. The hierarchy of this model ranged from a global level such as a health state utility, to HRQL domains, and to specific determinants of domains such as personality characteristics, [32] which may include adaptive abilities. Thus, the second aim of this study was to investigate if adaptive abilities affect health state utilities via HRQL domains.

Is the relation between adaptive abilities and health state utilities mediated by HRQL domains [33]?

Since we investigated psychological adaptive abilities we assume from a theoretical point of view that only mental health can mediate this relation.

## Methods

### Participants and design

We chose to study our research questions in a sample of patients with rheumatoid arthritis (RA). RA concerns a chronic disease with a wide spectrum of manifestations, for which adaptation is relevant, since no cure is available. From the database of the Leiden University Medical Centre, 300 people who were between 18 and 76 years old and had visited their treating rheumatologist in the previous six months were randomly selected. In total 1054 patients had visited their rheumatologist in the past six months. These patients were randomly numbered. First, 400 numbers had been drawn (using the software Excell) as a selection for a different study [34]. Of the remaining 654 patients 90 patients had to be excluded due to age restrictions, and 10 were excluded because they had refused to participate in a similar study [35]. Next, to get equal male/female distribution, 150 male patients and 150 female patients were randomly selected to participate in the current study. Based on the medical records, 50 people who had not been diagnosed with RA, and seven with severe co-morbid conditions were excluded. The remaining 243 eligible people received information about the survey by mail, including an informed consent form. Patients who did not return the informed consent form within three weeks were called as a reminder. Data were collected using self-report questionnaires and a semi-structured interview. The medical ethics committee of the Leiden University Medical Centre approved the study protocol.

### The interview

Face-to-face interviews were performed by three trained interviewers following a strict interview protocol. The interviews took place at the persons' preferred location; at home, in the hospital, or at work. A full description of the interview can be found elsewhere [36]. Here, only the part of the interview used to gather the information necessary for this study is described.

At the beginning of the interview, people valued their health of the previous week using a visual analogue scale (VAS) and a time tradeoff (TTO). Next people completed three questionnaires: the EQ-5D questionnaire [37], two scales of the Patient Satisfaction Questionnaire [38] and, the Rosenberg Self-Esteem Scale [39]. In this study only the information retrieved by the Rosenberg Self-Esteem Scale will be used. After the interview, people were asked to complete a questionnaire at home to lessen the burden. Among others this questionnaire included the Life Orientation Test [40], the Mastery scale of Pearlin and Schooler [41], and the MOS 36-item Short-From Health Survey (SF-36)[42].

### Instruments

#### The Visual Analogue Scale (VAS)

The VAS is a 100 mm horizontal line ranging from death to perfect health. Perfect health was described as full well-being in physical, psychological, and social functioning. Utility for the own health state of last week was elicited by asking respondents to place a mark between death and perfect health.

#### The Time tradeoff (TTO)

The computer program Ci3 [43] was used to elicit the TTO utilities based on a ping-pong search procedure. On the computer screen a short description of perfect health and a description of the patient's own health state of the previous week were presented. Perfect health was again described as full well-being in physical, psychological and social functioning. People rated how many years (x) of their remaining life expectancy (y), derived from Dutch life expectancy tables [44], they were willing to trade to obtain perfect health. Life expectancy was used as the time frame since it was shown to be more meaningful to the participant [45] and to lead to less loss aversion [46]. Utility was calculated as $\frac{\left(\text{y}-\text{x}\right)}{\text{y}}$.

### Indicators for persons' adaptive abilities

#### Personal Control

The Mastery List of Pearlin and Schooler [41] measures the extent to which people feel they are in control of their lives. People indicated their agreement with seven items such as "I can do about anything I really set my mind to do", on a five-point Likert scale ranging from 'totally disagree' to 'totally agree'. Total score ranged from 7-35, with a higher score indicating more control. Good internal consistency (alpha = .58 - .70) was reported previously among patients with a chronic illness [47].

#### Self-Esteem

With the Rosenberg self-esteem scale [39] the positive or negative valuation people have toward themselves was measured. People rated how much they agreed with 10 statements such as "I feel I have a number of good qualities", on a four-point Likert scale. The total score of the scale ranges from 0-30, with a higher score indicating higher self-esteem. Among patients with a chronic illness good internal consistency (alpha = .83 - .90) and test-retest reliability (*r *= .71) were reported previously [47,48].

#### Optimism

The Revised Life Orientation Test (R-LOT) [49] consists of three items measuring pessimism, three items measuring optimism and four filler items. Items such as "In uncertain times, I usually expect the best", were scored on a five-point scale ranging from 'strongly disagree' to 'strongly agree'. The total score, ranging from 0-24, was calculated after recoding items measuring pessimism. A higher score indicates more optimism. The R-LOT previously revealed good internal consistency (alpha = .74 - .89) and test-retest reliability (*r *= .67) among patients with a chronic illness [47,48].

#### Health related quality of life

HRQL was measured with the SF-36 [42]. The SF-36 comprises eight multi-item dimensions which can be summed into a physical and a mental component score (SF-36 PCS and SF-36 MCS). Scores in each component range from 0-100, with higher scores indicating better HRQL.

### Data Analysis

Prior to the main analyses, all variables were examined for uni- and multivariate outliers, missing data, linearity and normality. Missing data were excluded listwise. Principal component analysis was performed to check if the constructs 'personal control', 'self-esteem' and 'optimism' could be combined in one scale. The number of factors were decided upon by an eigenvalue > 1 and the scree plot. If the constructs measured one underlying factor, the standardized total scores of the separate constructs were summed and used as one scale measuring adaptive abilities. To further analyze the reliability of this scale Cronbach's alpha was calculated.

Hierarchical linear regression was conducted to assess if adaptive abilities could explain the variance in utilities above that explained by HRQL. To control for HRQL, the total scores on the PCS and MCS were entered first. In the next step the adaptive abilities were added. Separate analyses were performed for the VAS and TTO. Mediation analyses were performed as suggested by Baron & Kenny [50]. First we investigated if adaptive abilities affected mental health; second, the relation of mental health with health state utilities was investigated; third we investigated the direct effect of adaptive abilities on health state utilities without controlling for mental health, and finally we checked if after controlling for mental health the direct effect of adaptive abilities and health state utilities decreased (partial mediation) or even became zero (full mediation) [50]. When partial mediation was shown, the Sobel test statistic [51] was used to test the strength of the mediation.

## Results

### Participants

Of the 243 people selected, 132 people gave their approval to be interviewed (54%). No differences in age and time since diagnosis between responders and non-responders were found. Of the responders, one person with emotional problems, and two persons who were not able to speak and understand Dutch were not invited for the interview. Of the interviewed patients four were excluded; three people could not finish the interview due to cognitive or concentration problems, and one person returned the questionnaire after more then a month. All variables met the assumptions for linearity and normality, except for health state utility measured by the TTO (skewness = -1.36, SE = .22).

The interviews were administered by three trained interviewers (following a strict script), and took place at the LUMC (N = 83), at the respondent's home (N = 41) or at work (N = 1)). People were not hospitalized at the time of the interview. Persons interviewed at home had on average more health problems than persons interviewed in the LUMC based on the SF-36 PCS score. No interviewer effect was found on the answers patients gave. Table 1 presents the demographic information of the 125 people who were included.

**Table 1:** Characteristics of people with RA included in this study (N = 125)

	Mean (min-max)	SD	N (%)
Age	58 (29-75)	10.80	
Gender			
Female			60(48%)
Education^a^			
Nine years or less			38(31%)
Between 10 and 12 years			62(49%)
13 years of more			24(19%)
Children			
Yes			105(84%)
Marital status			
Married			110(88%)
Divorced/Widow			9 (7%)
Single			6 (5%)
Time since diagnosis (years)	13 (2 - 47)	9.26	
Health state Utilities			
VAS	66.14 (14 - 100)	19.15	
TTO	.77 (0 - 1)	.25	
Health status			
SF-36 PCS	36.46 (12-58)	10.66	
SF-36 MCS	52.36 (24-67)	9.66	

^a ^Numbers do not add up to 125 due to missing data.

### Creating a scale measuring persons' ability to adapt

Principal component analysis of the three indicators of persons' ability to adapt (Personal control, Self-esteem, and Optimism) could be aggregated to one component. This component explained 73% of the variance, the component loadings for self-esteem, personal control and optimism ranged from .81-.88. With reliability analysis the scale measuring persons' ability to adapt showed good internal consistency, Cronbach's alpha = .80.

### Predicting utilities

Before hierarchical regression analyses, the associations between the utility measures and demographic characteristics (time since diagnosis, gender, age, having a partner, having children, and education) and the study variables (PCS, MCS, and persons' ability to adapt) were checked with Pearson correlations. The demographic characteristics showed low to no correlation with the TTO and VAS (all *r *< .20). All study variables showed moderate to strong intercorrelation (table 2).

**Table 2:** Pearson correlations of study variables

	TTO	VAS
Persons' ability to adapt	.33**	.65**
SF-36 PCS	.30**	.57**
SF-36 MCS	.33**	.43**

* *p *< .05, ** *p *< .01

### Adaptive ability as direct predictor of TTO and the VAS, over and above HRQL

Table 3 presents the relationships of HRQL and persons' ability to adapt with utilities measured by the TTO and VAS, using a two-step hierarchical regression analysis. HRQL explained 19% of the TTO and 49% of the VAS. After correcting for HRQL, persons' ability to adapt did not predict additional variance in the TTO. On the VAS 5% additional variance was explained by persons' ability to adapt.

**Table 3:** Hierarchical regression analyses direct influence of adaptive abilities on TTO and the VAS above HRQL

Predictors		**Δ *R***^***2***^	*B*	β
TTO				

N = 123	*Step 1*	.192, *p *< .001		
	SF-36 PCS		.006	.265, *p *= .000
	SF-36 MCS		.009	.331, *p *= .000
	*Step 2*	.000, *p *= .886		
	SF-36 PCS		.006	.260, *p *= .006
	SF-36 MCS		.009	.319, *p *= .006
	Persons' ability to adapt		.000	.018, *p *= .886

VAS				

N = 123	*Step 1*	.487, *p *< .001		
	SF-36 PCS		.956	.529, *p *= .000
	SF-36 MCS		.848	.420, *p *= .001
	*Step 2*	.048, *p *= .001		
	SF-36 PCS		.761	.421, *p *= .000
	SF-36 MCS		.432	.214, *p *= .014
	Persons' ability to adapt		.441	.325, *p *= .001

Although persons' ability to adapt had no direct effect on health state utilities over and above the HRQL domains, it might have had an effect on HRQL domains that in turn affect health state utilities (mediation). Therefore this mediation effect was examined next. Firstly, it was found that persons' ability to adapt affected mental health, after correction of physical health (Δ *R*^2 ^= .46, *p *< .001). Secondly, mental health was related to health state utilities (Δ *R*^2 ^= .11, *p *< .01 for the TTO and Δ *R*^2 ^= .18, *p *< .01 for the VAS). Third, without correcting for mental health, persons' ability to adapt (Δ *R*^2 ^= .06, *p *< .001) did have a direct effect on health state utilities measured with the TTO and with the VAS (Δ *R*^2 ^= .20, *p *< .001). Finally, we found that the effect of persons' ability to adapt on both utility measurements decreased after controlling for mental health. As can be seen from table 3 (explained previously) persons' ability to adapt was completely mediated by mental health when health state utility was measured with TTO. The explained variance of VAS by persons' ability to adapt on VAS decreased from 20% to 5% when mental health was added, which was a significant change (Sobel test statistic [51] = 5.45, p <.001), indicating partial mediation.

## Discussion

In discussion sections of papers and in theoretical manuscripts, the difference in health state utilities between people with a chronic illness and the public is often explained by adaptation [1,14]. The results of this study show that adaptive abilities are indeed related to utilities, but that this effect is fully mediated by mental health for the TTO, and partly mediated for the VAS. It seems that in people with a chronic illness a stronger ability to adapt may lead to better mental health, which in turn leads to higher health state utilities. The suggested relation between adaptation and health state utilities given by people with a chronic illness does not occur directly, but appears to be mediated by mental health. Admittedly, this conclusion has to be made with caution since not adaptation but adaptive abilities are studied here.

Adaptive abilities explained 46% of the variance in mental health, which in turn explained between 11 - 18% of the variance in health state utilities after correction for physical health. Arnold et al. [52], already suggested such a mediation effect. They found that people with a chronic illness do not differ from healthy people in global quality of life and that global quality of life is mostly explained by mental functioning. Based on these findings they argued that people with a chronic illness psychologically adapt, causing a recovery of their mental health, which leads to recovery of global quality of life.

The cross-sectional design of this study limits the points described above. From this study no conclusions can be drawn about the causal relationship between persons' ability to adapt, HRQL, and health state utilities. Nevertheless, causal relations between persons' ability to adapt and HRQL have been described previously [24,47]. Future longitudinal research is necessary to further investigate this causal relationship.

The index based on CAT to measure persons' ability to adapt, has been used in several studies but has not yet been validated. Given the number of studies using such a scale based on the CAT, validation is pressingly needed. Further, this index has been suggested to reflect stable personality traits, which might not change over time [29]. If adaptive abilities are indeed stable over time, then health state utilities of members of the public might be influenced in a similar way. Yet since members of the public find it difficult to anticipate on their ability to adapt [11] we still would expect a less substantial effect of adaptive abilities on HRQL and health state utilities in this population.

HRQL predicted 20% of the variance in the TTO, and 49% of the variance in the VAS. These results are comparable with previous findings concerning the relationship between HRQL and health state utilities [53]. The smaller amount of variance explained in the TTO compared to the VAS might be caused by the trading process. In this trading process, a series of information processing activities and construction of subjective values for dimensions are developed, making the variance in TTO-scores difficult to explain. Another explanation may lie in the cognitive nature of the TTO. Campbell [54] suggested that quality of life can be assessed with cognitive or with affective measurements. Cognitive measurements depend on a more intellectual process while affective measurements depend on subjective feelings. The TTO might be seen as a more cognitive measurement, the VAS as a more affective measurement. After a life event, the affective component of well-being appears to be more impaired than the cognitive component, which means that this component is sensitive to change and the cognitive component is more stable [55]. Finally, a more methodological explanation for the smaller amount of variance explained in the TTO might be that the TTO was skewed. When a dependent variable is skewed a smaller effect size might be anticipated [56].

This study included patients with RA who had been diagnosed on average 13 years before. First, it can be questioned if patients still need to adapt to their illness so many years after diagnosis. It seems evident that adaptation takes place in the initial phase of the illness. However, the disabling, often progressive and uncontrollable characteristics of RA might result in adaptive processes, even after so many years. The results of this study indicate that adaptive abilities indirectly explain health state utilities, so this result might become more distinct when examining patients in the initial phase of their illness. Secondly, RA is a chronic illness characterized by pain and deformity of the joints, leading to physical limitations. There is evidence suggesting that people do not adapt to unpredictable stressors such as pain [57]. On the other hand, patients with RA might be able to adapt to other aspects of their illness such as the physical limitations by learning new ways to perform activities and they might learn to accept their pain [58]. More research is necessary to investigate the effect of adaptive abilities on health state valuations in other patient groups.

## Conclusion

In conclusion, the results in this study seem to indicate that adaptive abilities indirectly explain health state utilities. Assuming that these adaptive abilities induce adaptation, then cost-utility analyses could partly be founded on utilities shaped by adaptation. Such utilities will result in less room for improvement between the patient's own health condition and perfect health, leading to a lack of justification to treat an illness [9]. Based on this challenge, one could argue that members of the public should provide valuations instead, but these respondents are limited in their knowledge and experience compared to patients, and perhaps anticipate insufficiently to adaptation. The results of this study call for a discussion about if and how adaptation should be compensated for in cost-utility analyses, but first longitudinal research is necessary on the relation between health state utilities and adaptation, before decisions about compensations for adaptation can be made.

## List of abbreviations

CAT: Cognitive Adaptation Theory; HRQL: Health Related Quality of Life; SF-36: MCS Short-Form 36 Mental Component Scale; SF-36: PCS Short-Form 36 Physical Component Scale; TTO: Time Trade-off; VAS: Visual Analogue Scale.

## Competing interests

The authors declare that they have no competing interests.

## Authors' contributions

YP and TPMV were involved in acquisition of data. YP performed the analysis and YP, AVR and AMS took part in interpretation of the data. The first draft of the paper written by YP was revised by AVR and AMS. All authors gave final approval of the version published.
